# Activation of Interleukin-1 Beta in Arterialized Vein Grafts and the Influence of the -511C/T IL-1β Gene Polymorphism

**DOI:** 10.3390/jcdd6020020

**Published:** 2019-04-30

**Authors:** Ayumi Aurea Miyakawa, Thaiz Ferraz Borin, Luciene Cristina Gastalho Campos, Thais Girão-Silva, Joao Carlos Ribeiro-Silva, Luis Alberto Oliveira Dallan, Jose Eduardo Krieger

**Affiliations:** Heart Institute (InCor), University of São Paulo Medical School, São Paulo, SP 05403-000, Brazil; thaiz80@yahoo.com.br (T.F.B.); luciene.campos@gmail.com (L.C.G.C.); thaisgirao@yahoo.com.br (T.G.-S.); cribeiro_@live.fr (J.C.R.-S.); dcidallan@incor.usp.br (L.A.O.D.)

**Keywords:** human saphenous vein, interleukin-1β, vein graft, gene polymorphisms

## Abstract

The interleukin-1 family is associated with innate immunity and inflammation. The latter has been linked to the genesis of cardiovascular diseases. We, therefore, investigated whether interleukin-1 beta (IL-1β) is activated during arterialization of vein grafts. First, we examined the activation of IL-1β using the rat arterialized jugular vein serially sampled for up to 90 days. IL-1β expression increased 18 times on day 1 in the arterialized rat jugular vein and remained five times above nonarterialized vein levels for up to 90 days. Similarly, IL-1β expression increased early (1–5 days) in human vein graft autopsy samples compared with late phases (1–4 years). Activation was also detected in ex vivo arterialized human saphenous veins. Upon stratification of the results, we uncovered a T allele promoter attenuating effect in IL-1β activation in response to hemodynamic stress. Altogether, the results show that IL-1β is activated during arterialization of vein grafts in rats and humans, and this response is modulated by -511C/T IL-1β gene polymorphism. It is tempting to speculate that the activation of IL-1β, and consequently local inflammation, modulates early vascular remodeling and that the gene polymorphism may be useful in predicting outcomes or assisting in interventions.

## 1. Introduction

Autologous coronary artery bypass graft (CABG) surgery is a common procedure used for revascularization in ischemic heart disease. Although arterial vessels are the preferred conduit, the saphenous vein is still widely used for this purpose. The limitation of saphenous vein grafts is the accelerated atherosclerosis and subsequent occlusion occurring at a rate of about 50% at 10 years after implantation [1]. Several aspects of the atherogenesis process have been described and involve complex interaction of inflammatory cells and vascular cells [2,3,4,5]. In this context, interleukin-1 beta (IL-1β) plays an important role as a pro-inflammatory cytokine and as the stimulator of the production of adhesion molecules, matrix metalloproteinases, growth factors, cytokines, and chemokines [6,7]. IL-1β can be synthesized and released by inflammatory cells, endothelial, and smooth muscle cells, and it alters vascular function and mediates vascular injury [6,8,9]. 

Recently, the first results of CANTOS (Canakinumab Anti-Inflammatory Thrombosis Outcome Study) showed that a monoclonal antibody targeting IL-1β in post-myocardial infarction patients reduces non-fatal myocardial infarction, non-fatal stroke, and cardiovascular death [10]. Later, researchers showed that the reduction of the risk of cardiovascular events is proportional to the reduction of the inflammatory response [11]. This evidence highlights the importance of inflammation in the development of cardiovascular complications.

The role of IL-1β is well documented in arterial lesions and in vein graft disease in both human and animal models [12,13]. In this work, we carefully demonstrate the time course activation of IL-1β upregulation using an in vivo rat model. We also validated this observation in samples from arterialized human saphenous vein and autopsy samples and provide evidence for the influence of-511C/T IL-1β polymorphism in IL-1β expression in the response to increased hemodynamic stress.

## 2. Materials and Methods

### 2.1. Rat Jugular Vein Arterialization 

Jugular vein arterialization was performed in our laboratory by arteriovenous connection via end-to-end anastomosis of the carotid artery and jugular vein as has been previously reported [14]. Transverse sections (3 μm-thick) were analyzed starting 400 μm from the suture. This study used no more than 50 μm of the total length of the arterialized vein. 

### 2.2. Human Saphenous Vein 

Human saphenous vein grafts were selected from autopsies performed at the Heart Institute (InCor), University of São Paulo Medical School. Samples derived from individuals who had died of a cardiovascular event were excluded from the study. According to the medical records, the cases were classified into two groups: early (1–5 days of saphenous vein graft surgery) and late (1–4 years of saphenous vein graft surgery). The middle third-part of each selected vein graft was analyzed by immunohistochemistry for IL-1β.

Fresh saphenous vein was obtained from patients undergoing coronary artery bypass graft surgery at the Heart Institute (InCor), University of São Paulo Medical School. The vein segment was kept in a physiological solution until mounting in the culture system. This study protocol was approved by the local ethics committee (SDC—1834/01/22, CAPPesq—299/01).

### 2.3. Ex Vivo Culture of Human Saphenous Vein 

Human saphenous veins were cultured using a flow-through system established in our laboratory [15]. Vein segments were placed in the chamber filled with Dulbecco’s modified Eagle’s medium containing 10% fetal bovine serum. Segments from the same patient were connected to the perfusion system and cultured for 24 hours either under venous condition (flow: 5 mL/min) or arterial condition (flow: 50 mL/min, pressure: 80 mmHg). All culture solutions were purchased from Invitrogen (Thermo Fisher Scientific, Waltham, MA, USA).

### 2.4. IL-1β Polymorphism Determination

Genomic DNA was extracted from peripheral blood leukocytes by the standard salting-out procedure. Analysis of -511C/T IL-1β polymorphism was performed as previously described [16]. The primers used for the polymerase chain reaction (PCR) were: 5′ TGGCATTGATCTGGTTCATC 3′ (forward) and 5′ GTTTAGGAATCTTCCCACTT 3′ (reverse).

### 2.5. Gene Expression Analysis

Total RNA was isolated with TRIzol Reagent according to the manufacturer’s instructions (Invitrogen Corporation, Carlsbad, CA, USA). cDNA synthesis was performed with random hexamers (High Capacity cDNA Archive kit—PE Applied Biosystems), and 5 ng of cDNA was used for real-time RT-PCR (reverse transcription polymerase chain reaction) reaction (SYBR^®^ Green PCR Master Mix—PE Applied Biosystems). All samples were assayed in triplicate, and 28S ribosomal RNA was used as an internal control. The comparative CT (threshold cycle) method was used for data analyses (User Bulletin #2). The primer sequences used were: 

rat IL-1β: 5′ TGAAGCAGCTATGGCAACTG 3′ (forward) and 5′-ATCTTTTGGGGTCTGTCAGC-3′ (reverse); human IL-1β: 5′-TCTGTACCTGTCCTGCGTGTTG-3′ (forward) and 5′-TTCTTTGGGTAATTTTTGGGATCT-3′ (reverse); 28S: 5′-TCATCAGACCCCAGAAAAGG-3′ (forward) and R: 5´-GATTCGGCAGGTGAGTTGTT-3′ (reverse).

### 2.6. Immunohistochemistry

Tissue sections of 3 μm were incubated with monoclonal anti-smooth muscle α-actin (clone 1A4) antibody (32.4 ng/mL, Sigma Aldrich, St. Louis, MO, USA) followed by the secondary antibody (Kit LSAB HRP Universal-DAKO). IL-1β detection was conducted by incubation with polyclonal anti-IL-1β (5 ng/mL, Santa Cruz Biotechnologies, Dallas, TX, USA) and developed with Fast Red (Vector Laboratory, Burlingame, CA, USA). The cross-sections were examined by light microscopy, and α-actin was visualized in brown and IL-1β in red.

### 2.7. Statistical Analysis

Data are expressed as means ± SEM. Statistical analyses were performed using one-way ANOVA. When only two groups were compared, the unpaired Student *t* test was carried out. Values of *p* < 0.05 were considered statistically significant.

## 3. Result

**Figure 1:**
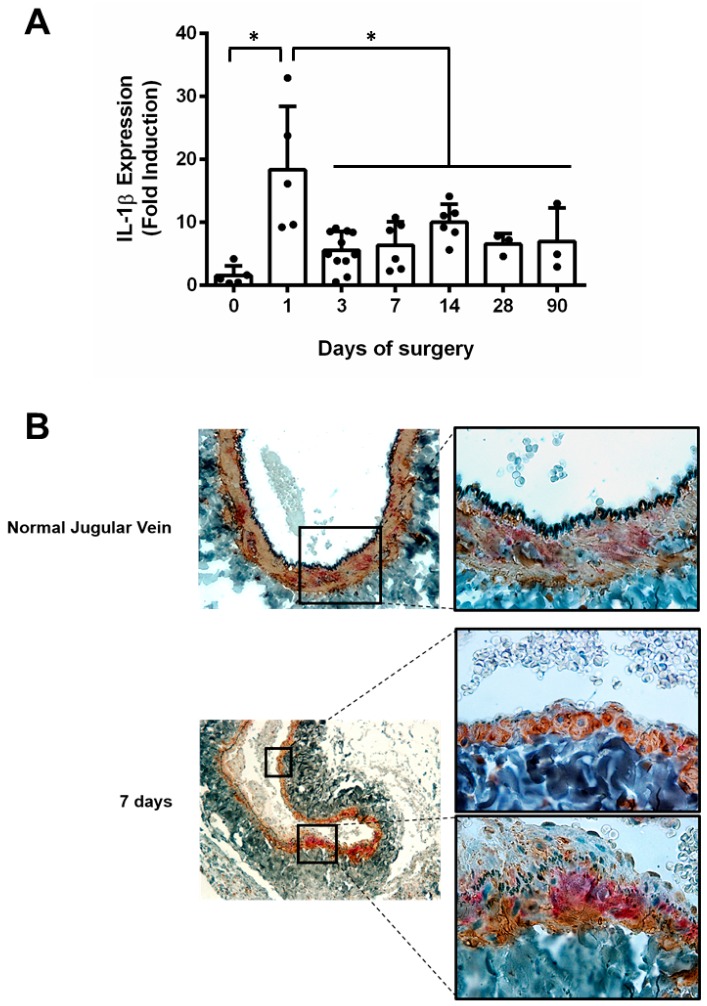
(**A**) interleukin-1 beta (IL-1β) expression in rat arterialized jugular vein. Real-time RT-PCR in arterialized jugular vein up to 90 days. The experiment was normalized by 28S rRNA, and each bar represents mean±SD of 3 to 11 experiments. * indicates *p* < 0.05. (**B**) Immunohistochemistry for IL-1β (red) and smooth muscle α-actin (brown) in arterialized jugular vein. Representative sections of normal jugular vein and arterialized jugular vein at 7 days.

The time course expression of IL-1β was evaluated during arterialization of the rat jugular vein for up to 90 days. After arterialization, expression of IL-1β increased 18 times on day 1, followed by a decrease from day 3 onwards, remaining about 5 times higher than the expression in the normal jugular vein (Figure 1A). Immunostaining revealed that IL-1β pattern of expression occurs in patches all over the normal jugular vein. After arterialization surgery, the expression of IL-1β is concentrated at regions of hyperplasia (Figure 1B). 

**Figure 2:**
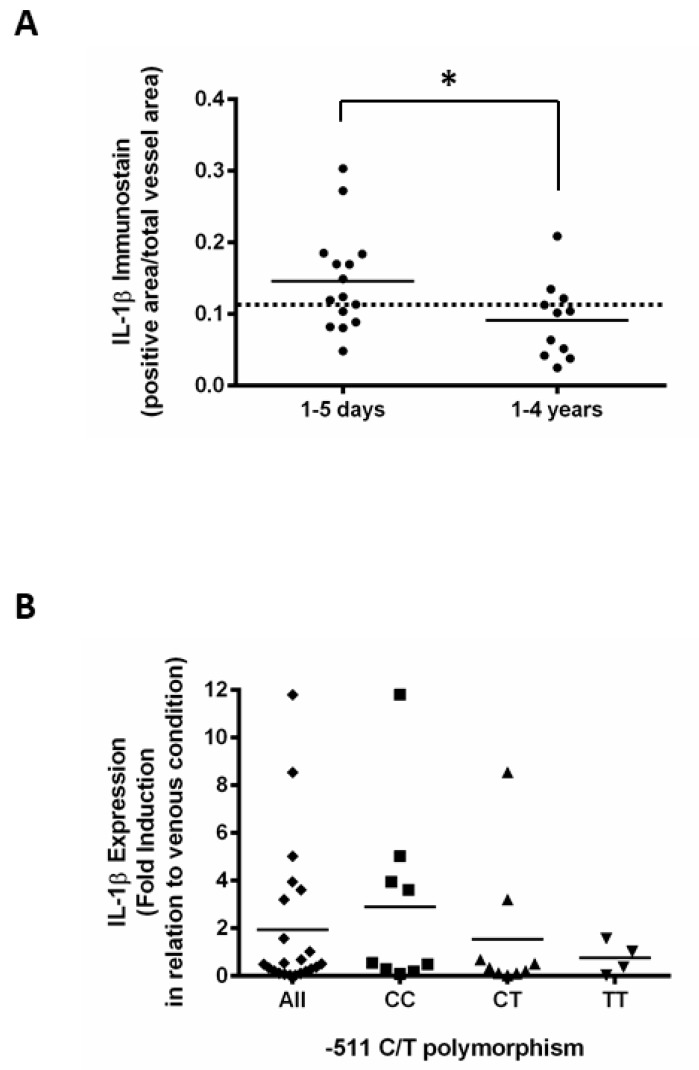
(**A**) Semi-quantitative analyses of IL-1β in a human saphenous vein graft obtained from autopsy. Immunohistochemistry for IL-1β was performed, and the positive-stained area was normalized by the total vessel area. Samples were grouped as early (1–5 days, N = 15) and late (1–4 years, N = 11) vein grafts. Fresh isolated human saphenous veins (N = 10) were used for reference (dashed line). * indicates *p* < 0.05. (**B**) Expression of IL-1β in arterialized human saphenous vein and evaluation of the influence of -511C/T IL-1β polymorphism. Real time RT-PCR for IL-1β was performed in human saphenous vein cultured in venous or arterial conditions. The results were normalized by 28S ribosomal RNA. Each dot represents the fold induction of the arterial sample compared with the venous sample. The graphic shows the results of all samples analyzed (♦) and the sample stratified by -511C/T IL-1β polymorphism. All (♦, N = 22), CC (■, N = 9), CT (▲, N = 9), TT (▼, N = 4).

To further verify whether IL-1β modulation also occurs in human samples, we performed immunohistochemistry with human saphenous vein grafts obtained from autopsies in which IL-1β increased in early (1–5 days) vein graft samples compared with late (1–4 years) vein graft samples that were similar to fresh non-arterialized saphenous vein samples (Figure 2A). This finding agrees with data obtained from an ex vivo culture system where exposure of human saphenous vein under arterial conditions for 24 hours resulted in a 2.7-fold induction of IL-1β expression compared with the venous condition (Figure 2B). 

Interestingly, we found that the T allele attenuated the activation of the IL-1β expression in response to hemodynamic stress when samples were stratified by the polymorphism at position -511 of the IL-1β promoter. We verified a 4.3-fold induction of IL-1β expression for the genotype CC compared with a 1.6-fold induction for the CT (N = 9) and TT (N = 4) genotypes (*p* < 0.05) (Figure 2B). It suggests that the activation of IL-1β expression in vein grafts exposed to hemodynamic stress is modulated by the -511C/T IL-1β polymorphism.

## 4. Discussion

Using a combination of in vivo and ex vivo vascular methods, we provide evidence that IL-1β is modulated in arterialized vein segments of animal and human samples and that an IL-1β genetic polymorphism modulates this response, which may be useful for predicting outcomes or to assist interventions to modulate the early inflammatory response associated with vein graft arterialization in the future. A great body of evidence suggests that IL-1β is regulated in vein graft exposed to arterial circulation using experimental models [8,17]. Here, we provide a detailed time-response for the activation of IL-1β expression, which shows an early peak on day 1 and elevated levels for at least 90 days. This profile is consistent with the idea of its role in early vein graft intimal hyperplasia because IL-1β production is localized to the developing neointima.

Data from saphenous vein graft obtained at autopsy suggest that the same pattern of regulation observed in the rat model might also be present in humans because 1- to 5-day autopsy samples showed increased levels of protein expression compared with 1- to 4-year samples that showed a level of protein similar to fresh non-arterialized samples. Additionally, by using an ex vivo experimental approach we showed that hemodynamic stress can increase IL-1β expression compared with a vein segment under venous conditions. Our data also indicate that vascular activation of IL-1β expression is influenced by the -511C/T IL-1β polymorphism. It has been demonstrated that the IL-1β gene polymorphism correlates to IL-1β secretion [18,19], and it is an indication that inter-individual differences in inflammatory response are related to genetic variation. Considering that IL-1β polymorphism might modulate an inflammation-triggered pathway, the implication of -511C/T IL-1β polymorphism has been studied in myocardial infarction, ischemic stroke, hypertension, atherogenesis, and thrombosis [19,20,21]. The understanding of the influence of this polymorphism and its potential predictive role in arterial-vein graft outcomes may help the use of post-surgery or in-surgery anti-inflammatory drug therapy, such as human monoclonal antibodies targeting IL-1β. This may be particularly important in light of the recently published trial, the Canakinumab Anti-inflammatory Thrombosis Outcome Study (CANTOS), a randomized, double-blind placebo-controlled trial that has shown that reducing inflammation among heart attack patients reduces the risk of another cardiovascular event: myocardial infarction, stroke, and cardiovascular death [11,22].

It is known that the intensity of inflammation and its timing in relation to vascular injury modulate inflammatory reaction and determine the progression of vascular repair [3,4,23]. Low-intensity or high-intensity inflammation seems to lead to the opposite effect on vascular remodeling, and we show in this study that IL-1β levels are increased in early stages of the arterialized vein. More recently, the Gary Owens group demonstrated that IL-1β is important in regulating the fibrous cap formation in advanced atherosclerotic lesions and suggested that excessive inhibition of inflammation have prejudicial effects [24,25]. This highlights the importance of understanding the dynamics of IL-1β expression to better design an effective therapeutic approach. It may not be a binary strategy and should take into account when the treatment is being performed (timing) and the amount of IL-1β in the lesion to be treated (levels). Considering that -511C/T IL-1β polymorphism influences the level of IL-1β, it can be valuable information to help guide the therapeutic strategy.

The CABG Genomic Program has been conducted to identify genetic causes of adverse events after cardiac surgery, and variants of chromosomes 9p21 and 4q25 were identified as predictors of mortality and atrial fibrillation after coronary artery bypass graft surgery [26,27]. Further studies should be conducted to evaluate whether the -511C/T IL-1β polymorphism can add value to predicting the outcome of vein graft patency and to the logistic EuroSCORE, a method of calculating predicted operative mortality for patients undergoing cardiac surgery [28,29,30]. In the era of personalized medicine, the more information a physician can have to assist the patient the better the treatment outcome.

In summary, we provide here evidence that IL-1β increases in arterialized rat jugular vein and human saphenous vein grafts. The early upregulation of IL-1β in response to increased hemodynamic conditions is influenced by the -511 C/T IL-1β polymorphism with the T allele conferring an attenuated response to hemodynamic stress.
